# Heat stress effects on milk yield traits and metabolites and mitigation strategies for dairy cattle breeds reared in tropical and sub-tropical countries

**DOI:** 10.3389/fvets.2023.1121499

**Published:** 2023-07-07

**Authors:** Vincent Habimana, Athumani Shabani Nguluma, Zabron Cuthibert Nziku, Chinyere Charlotte Ekine-Dzivenu, Gota Morota, Raphael Mrode, Sebastian Wilson Chenyambuga

**Affiliations:** ^1^Department of Animal, Aquaculture, and Range Sciences, Sokoine University of Agriculture, Morogoro, Tanzania; ^2^SACIDS Africa Centre of Excellence for Infectious Diseases, SACIDS Foundation for One Health, Sokoine University of Agriculture, Morogoro, Tanzania; ^3^International Livestock Research Institute (ILRI), Nairobi, Kenya; ^4^Tanzania Livestock Research Institute (TALIRI) Eastern Zone, Tanga, Tanzania; ^5^School of Animal Sciences, Virginia Polytechnic Institute and State University, Blacksburg, VA, United States

**Keywords:** heat stress, milk yield and composition, milk and blood metabolites, physiological parameters, tropical countries, mitigation strategies

## Abstract

Heat stress is an important problem for dairy industry in many parts of the world owing to its adverse effects on productivity and profitability. Heat stress in dairy cattle is caused by an increase in core body temperature, which affects the fat production in the mammary gland. It reduces milk yield, dry matter intake, and alters the milk composition, such as fat, protein, lactose, and solids-not-fats percentages among others. Understanding the biological mechanisms of climatic adaptation, identifying and exploring signatures of selection, genomic diversity and identification of candidate genes for heat tolerance within indicine and taurine dairy breeds is an important progression toward breeding better dairy cattle adapted to changing climatic conditions of the tropics. Identifying breeds that are heat tolerant and their use in genetic improvement programs is crucial for improving dairy cattle productivity and profitability in the tropics. Genetic improvement for heat tolerance requires availability of genetic parameters, but these genetic parameters are currently missing in many tropical countries. In this article, we reviewed the HS effects on dairy cattle with regard to (1) physiological parameters; (2) milk yield and composition traits; and (3) milk and blood metabolites for dairy cattle reared in tropical countries. In addition, mitigation strategies such as physical modification of environment, nutritional, and genetic development of heat tolerant dairy cattle to prevent the adverse effects of HS on dairy cattle are discussed. In tropical climates, a more and cost-effective strategy to overcome HS effects is to genetically select more adaptable and heat tolerant breeds, use of crossbred animals for milk production, i.e., crosses between indicine breeds such as Gir, white fulani, N’Dama, Sahiwal or Boran to taurine breeds such as Holstein-Friesian, Jersey or Brown Swiss. The results of this review will contribute to policy formulations with regard to strategies for mitigating the effects of HS on dairy cattle in tropical countries.

## 1. Introduction

Heat stress is a condition in which animals become unable to dissipate heat load generated from their body metabolism and environment, thereby failing to maintain their body thermal balance (1–3). Heat stress leads to an increase in rectal temperature (RT), respiration rate (RR), core-body temperature (CBT), panting score (PS), pulse rate (PR), sweating rates (SR), and heart rate (HR) (4). Heat stress has adverse effects on milk production traits, fertility and heath in dairy cattle (5), especially in tropical countries where the climate is always hot and humid (6). Dairy cattle in these regions are exposed to higher ambient temperature (Ta), relative humidity (RH), solar radiation (7) and wind variations for extended periods of time. In dairy cattle, temperature and humidity, usually combined as temperature-humidity index, (THI) surpassing their thermoneutral zone cause high reduction in dry matter intake, milk yield, and fertility, which are associated with physiological changes including CBT, RR, PR, RT, and SR (8, 9). These effects on milk production and composition, including fat, protein, lactose, and solids-not-fat percentages, can directly impact the income generated from dairy farming (10). It is crucial for dairy cattle to be within its thermoneutral zone, where energy expenditure to maintain internal body temperature regulation is not required (3). The thermoneutral zone for *Bos taurus* dairy cattle breeds typically ranges from 0.5°C to 20°C (8), although this can vary depending on factors such as production status, acclimatization level, pregnancy status, feed type, and climatic conditions including wind speed, solar radiation, and relative humidity (3, 8).

Even in countries with moderate temperature such as in the European Union (EU) and the United States of America (USA) dairy cattle can still experience heat stress (HS) due to the effect of climate on evaporative cooling (3). Heat stress has been shown to cause significant economic losses in the dairy industry, with annual losses ranging from 1.69 to 2.26 billion dollars in the USA and a reduction of 0.73 million liters of milk in India in 2020. In the United Kingdom (UK), where the majority of dairy herds are composed of Holstein-Friesian breeds which are known for high milk yield, the projected annual average milk yield loss is 2.4% (~170 kg/cow) in the South West regions (3). Genetic selection for heat tolerance in dairy cattle without compromising their milk yield potential is seen as a sustainable strategy to support dairy cattle production in tropical environments. Dairy cattle breeds in tropical countries are diverse, including heat-adapted *Bos indicus* cattle and less resilient *Bos taurus* breeds, and they may respond differently to the effects of HS due to their genetic makeup (11). Therefore, genetic evaluation for thermotolerance and selection of heat-tolerant animals based on specific regions and climates is possible and is a cost-effective measure to improve dairy cattle production in hot weather conditions. It should be noted that milk production traits are often antagonistic to heat tolerance, and genetic selection for higher milk production without considering heat tolerance may result in increased susceptibility to HS (4, 12, 13). Therefore, a balanced approach that considers both milk production traits and heat tolerance is necessary for effective genetic selection in dairy cattle breeding programs. The study by Dikmen et al. (14) reported that heat-tolerant Holstein cows had higher milk yield and better reproductive performance compared to heat-susceptible cows in a tropical environment. Sigdel et al. (15) found that crossbreeding Holstein cows with heat-tolerant breeds such as Sahiwal and Gir improved milk yield and reproductive performance under HS conditions. Furthermore, Ekine-Dzivenu et al. (13) demonstrated that incorporating thermotolerance as a selection criterion in breeding programs can improve milk production.

In addition to genetic selection, environmental modifications can also be implemented to mitigate the impact of HS on dairy cattle. These may include providing shade, improving ventilation, using evaporative cooling systems, and adjusting feeding and watering practices. However, these measures may not always be cost-effective or feasible, especially in resource-limited tropical environments (4, 6). Therefore, integrating genetic selection for heat tolerance into breeding programs can be a more sustainable and long-term solution to mitigate the adverse effects of HS on milk production traits, fertility, and health in dairy cattle. Therefore, the objective of this review was (1) to characterize the genetic and phenotypic responses to HS, (2) identify candidate genes responsible for heat tolerance; and (3) discuss mitigation strategies for dairy cattle breeds reared in tropical countries. The findings of this review will contribute to policy formulations with regard to strategies for preventing the effects of HS on dairy cattle breeds in sub-Saharan Africa, where the climate is very hot and humid almost year-round.

## 2. Dairy cattle breeds reared in tropical countries

Cattle breeds used for milk production in Africa are categorized into four groups: the humpless *Bos taurus,* found mostly in west Africa; the humped *Bos indicus* (Zebu), temperate taurine, which have been disseminated extensively in Africa; Sanga which is a crossbred of *B. taurus* x *B. indicus*, predominantly reared in eastern and southern Africa; and Sanga x Zebu cross well-known as Zenga (16, 17). The dominant cattle breed reared in Africa is the Zebu cattle, which is effectively adapted to the tropical climates and tolerant to extreme temperatures and HS. Moreover, the breed is highly resistant to diseases and parasites like ticks and has low nutritional requirements (18). However, the breed has low potential for milk production.

In Sub-Saharan Africa (SSA), breeds commonly used for dairy production are mainly crossbreds from local breeds and imported animals or semen from USA, Canada, Europe, Australia and New Zealand. Holstein is the preferred exotic breed for milk production in SSA, followed by Jersey, Ayrshire and Guernsey (19, 20). Among the dairy cattle breeds introduced in Africa, the Holstein dairy cattle breed is the predominant breed in most countries and it is the more productive genotype worldwide (21). Additionally, the Holstein genotype is more favored by farmers due to its ability for high milk production, which lead to high profits at the farm. Jersey, Ayrshire and Guernsey genotypes are considered as lower milk producers and are, then, less preferred. On the other hand, Jerseys are preferred owing to the high butterfat content of their milk and the relatively lower rearing costs due to their small size (20). It is well known that the Holstein and Jersey breeds differ in their abilities to respond to HS effects. Many studies have indicated that the Jersey breed is more tolerant to HS compared to Holstein dairy cattle based on milk production decline (19).

Because of lack of adaptability of pure dairy cattle breeds to tropical environment, several countries in sub-Saharan Africa have resorted to using crosses of Holstein-Friesian, Ayrshire, Jersey, with *Bos indicus* for milk production. Crossbreeding of indigenous cattle with exotic dairy breeds has been utilized to improve milk yield in hot regions and attaining adequate reproduction rates. Currently, there are a large number of crossbreds produced by crossing the exotic dairy genotypes and local Zebu cattle which are used as dairy cows (19) in sub-Saharan countries. In most tropical countries crossbreeding has been used to combine high production traits of *B. taurus* (Holstein) with greater heat tolerance of *B. indicus* (Zebu). In some countries, this strategy has led to development of a higher producing and heat-tolerant genotype such as Girolando genotype in Brazil (22). In Thailand, crossbreeding between *Bos taurus* (Holstein, Jersey, and other dairy cattle breeds) and *Bos indicus* (Sahiwal, Red Sindhi, and Thai Native) is predominant and the main crossbred dairy cows (>87.5% Holstein genetic composition) are crosses of Holstein and Sahiwal or Thai Native breeds (23). Studies in tropical countries have indicated that Holstein-Friesian crossbreds have relatively better milk production performance while Jersey crossbreeds have higher fertility in comparison to the anterior (24). In addition to crossbreds, *Bos indicus* genotypes, e.g., Sahiwal and Gir, which are regarded to be highly resilient to tropical environment and slightly sensitive to HS are used for milk production (25). Table 1 shows different dairy cattle breeds reared in tropical countries for milk production.

**Table 1:** Dairy cattle breeds reared in tropical countries.

Region	Country	Dairy cattle breeds	References
East Africa	Ethiopia, Somalia	Horro, Guraghe, Arsi, Abigar, Zebu, Boran, Ambo, Adwa, Ogaden, Zebu Sanga, Fogera, Sanga, Raya-Azebo, Kerayu, Danakil, Bonga, Jimma, African taurine Sheko, East African shorthorn Zebu and Barca	Galukande et al. (26), Bora et al. (27)	Burundi, DRC, Kenya, Rwanda, South Sudan, Uganda, Tanzania	Ankole, Holstein, Friesian, Jersey, Ayshire, Sahiwal, Boran, and East African shorthorn Zebu	Rewe et al. (28), Kabi et al. (29)	Tanzania	Boran, Sahiwal, Friesian, Tanzania Shorthorn Zebu	Msalya et al. (30), Kibona et al. (31)
West and central Africa	Nigeria, Cameroon	*Bos taurus* (humpless shorthorns and longhorns), White Fulani	Mwai et al. (32), Norezzine et al. (33)	Chad, Sudan, Senegal	White Fulani, Zebu Fulani	Norezzine et al. (33)
Southern Africa	South Africa, Zambia	Sanga cattle, Angoni, Tonga, Barotse, Mambwe, Baila and Lenje, Nsenga, Lozi, Swaka, Boran, Afrikander, Bonsmara, Jersey, Friesian, and Ayrshire	Mwai et al. (32), Odubote (34)
North Africa	Algerie	Brown Atlas, Montbeliarde, Brune des Alpes	FAO (35)	Egypt	*Bos taurus*, Brown atlas breed	FAO (35)	Mauritania	Moorish zebu and Fulani zebu	FAO (35)	Morocco	Brown atlas breed	FAO (35)	Tunisia	Normandy, Eastern Red and White, Brown Suiss, Tarentaise	FAO (35)
Asia	India	Ayshire, Brown Suiss, Guensey, Holstein, Friesian, Jersey, Sahiwal, Gir, Guzerá, Ratini, Kankrej, Tharparkar, Red Sindhi, Ongole, Deshi and Hariana	Saravanan et al. (36), Velayudhan et al. (37), Worku et al. (38), Peixoto et al. (39), Verma et al. (40)	Bangladesh, Thailand	Sahiwal, Holstein Friesian, Brown Suiss, Jersey, Red Dene, Red Sindhi, Brahman, and Thai Native	Chanda et al. (41), Boonkum et al. (6)	Sri Lanka	Deshi, Sindi, Sihaha	Chanda et al. (41)
Latin America	Costa Rica, Brazil	Criollo, Creole, Guzerá, Junqueira, Franqueiro, Caracu, Mocho Nacional, Nellore, Jersey, Gir, Holstein, Girolando	Egito et al. (42), Santana et al. (43), Santana et al. (44), Stumpf et al. (22)

## 3. Indicators and phenotypic responses to heat stress for dairy cattle breeds reared in the tropics

The indicators of the impact of HS in animals include: (1) physiological parameters (e.g., respiration rate, rectal temperature, core-body temperature, sweating rate, panting score, among others); (2) milk production traits; (3) oxidative stress biomarkers; and (4) genes influencing HS tolerance. Additionally, the THI which combines the effects of ambient temperature and relative humidity, wind speed and solar radiation is also a proxy for HS conditions, and may approximate potential HS in dairy cattle. A comprehensive review about those HS biomarkers was presented in our previous review (45). Table 2 shows indicators of HS in dairy cattle and methods used to measure them.

**Table 2:** Indicators of heat stress in dairy cattle.

Indicator		Methodology	References
Climate variables	Ambient temperature, relative humidity, wind speed, solar radiation, THI	Collected at public weather stations	Bohmanova et al. (46), Carabaño et al. (47)
Phenotypic traits	Core-body temperature	Measured using infrared thermometer	Osei-Amponsah et al. (9)
	Rectal temperature	Measured using clinical veterinary thermometer inserted at 30 cm in the rectum wall of dairy cattle for 1 min	Dalcin et al. (48)
	Respiration rate	Measured using a stethoscope and a stopwatch for 15 s, then multiply the value obtained to get the breaths per min. Measured also through counting the right thoracoabdominal movements for 30 s and multiply the value obtained by two to get the breaths per min.	Dalcin et al. (48), Osei-Amponsah et al. (9), Pinto et al. (49)
	Panting score	Measured by visual observation in a scale of 0 to 4	Mader et al. (50)
	Drooling score	Visual observation checking saliva coming out of the mouth when dairy cattle is not ruminating	Mader et al. (50)
	Sweating rate	Visual observation	Osei-Amponsah et al. (9)
	Heart rate	Measured using a stethoscope and a stopwatch for 30 s, then multiply the value obtained to get the beats per min	Dalcin et al. (48)
	Pulse rate	Measured using stethoscope	Berian (51), Gaafar et al. (52)
Milk yield trait	Milk yield decline	Recorded immediately after hand milking or automatic milking system	Li et al. (53)
Milk quality traits	Fats percentage	Measured using Lactoscan/ mid infrared spectroscopy	Zheng et al. (54), Honig et al. (55)
	Protein percentage		
	Lactose		
	Solids-not-fats		
	Somatic cell count	Lactoscan, California Mastitis Test, deLaval cell counter	Lambertz et al. (56), Persson and Olofsson (57)
	Somatic cell score		
Animal behaviors	Grazing, water and feed intake, rumination, lying down	Visual observation while dairy cattle are grazing at farm and pedometer	Reis et al. (58)

Dairy cattle have developed three coping mechanisms, i.e., acclimation, acclimatization, and adaptation to reduce the impacts of HS on their biological systems (59, 60). Acclimation is the coordinated phenotypic response developed by dairy cattle to a particular environmental stressor while acclimatization is a coordinated response to numerous simultaneous stressors such as temperature, humidity, wind speed, and solar radiation (59, 60). Both acclimation and acclimatization act to improve dairy cattle fitness to the changing climatic conditions (59). On the other hand, adaptation involves genetic changes as negative effects of environmental variables on an animal persist over long time (59, 60). To cope with HS conditions, dairy cattle spend long time standing and reduce activity to rise surface area for heat abatement, sensible water loss, radiating surface area, and air movement via convection, conduction, radiation, and evaporation (61, 62). Heat stress prevents dairy cattle to fully express their genetic potentials for milk yield and affects milk composition (63). Dairy cattle react to HS through: (1) decline in feed intake, (2) high water consumption, (3) altered basal metabolic rate, (4) increased evaporation of water, (5) physiological changes, i.e., higher body temperature, higher respiratory rate, panting rate, pulse rate, heart rate, rectal temperature, and (6) altered blood hormone concentration (45). A dairy cow experiencing HS produces small quantity of milk and has a high risk of disease infection (61). Under HS conditions, the decrease in feed intake has been recognized as an important cause of decline in milk production as it is associated with a negative energy balance state regardless of the lactation stage (41). Feed intake in lactating Holstein cows begins to decline at the ambient temperatures of 25–26°C and decreases sharply when the temperature is over 30°C. At 40°C, feed intake can decrease by 40% (41).

Heat stress is associated with reduced milk, fat, and protein yields (64–66). For instance, Negri et al. (67) reported 21% milk yield losses in a commercial herd of Holstein dairy cattle reared in southern part of Brazil due to HS. In Bangladesh, Chanda et al. (41) indicated that when a lactating Holstein cow is moved from an ambient temperature of 18 to 30°C milk yield is decreased by around 15%, followed by a 35% decline in the efficacious of energy usage for production reasons.

The THI is used to quantify environmental heat load on animals. Milk yield is negatively associated with THI. In Thailand, Sungkhapreecha et al. (23) found a smaller decrease in milk production of about 70–80 g per point increase in THI value of 76 for cows with >93.7% Holstein genetic composition, and a 20–40% lower milk production in comparison to other populations. In this study, crossbred dairy cattle >87.5% Holstein genetics were crossbreeds between Holstein and Sahiwal or Thai Native breeds (23). Moreover, milk yield, milk fat, solids-not fat, and protein percentages are reduced by 15, 39.7, 18.9, and 16.9%, respectively, when a lactating Holstein cow is transferred from an ambient temperature of 18 to 30°C (68). Table 3 presents information about dairy cattle phenotypic responses to HS for studies conducted in tropical countries of Asia, Africa and Latin America.

**Table 3:** Phenotypic responses to heat stress for dairy cattle breeds reared in tropical countries.

Region	Country	Dairy cattle breed	Phenotypes	References
Asia	India	Sahiwal, Hariana	Milk yield decline at THI value of 60 (winter) due to a decline in minimum temperature. Higher respiratory rate (RT), rectal temperature (RT), and pulse rate (PR) at THI value of 86 (summer)	Kumar et al. (69)		Holstein	Milk yield decline and alteration of body condition score at THI value of 75	Velayudhan et al. (70)		Holstein, Jersey	THI values ranged from 67.43–86.92 with threshold at 75.77. There was a higher RR of 29.66 breaths/min in the summer compared to 23.44 breaths/min in the monsoon. At THI ≥75, surface body temperature was 32.21°C compared with 29.92°C at THI<75.	Velayudhan et al. (37)		Holstein	Milk yield decline from 18 ± 1.4 to 10 ± 0.92 L at THI value above 72 from June–October.	Kohli et al. (71)		Holstein Hariana	Higher RR and RT at THI values of 74, 80 and 82.	Jeelani et al. (72)	Thailand	Holstein	Milk yield losses (THI = 72–82) estimated at 0.10Kg and 0.06Kg/unit of THI increase for Holstein dairy cows below 93.7% and higher than 93.7% blood level	Sungkhapreecha et al. (5)	Bangladesh		Milk yield decline of 0.26 kg/day for every rise in THI value above 84	Chanda et al. (41)	Sri Lanka		Milk yield decline at 0.8Kg/day for every unit rise in THI value above 80	Narmilan et al. (73)
Africa	Rwanda	Ankole, Holstein, and Jersey	Milk yield declined at THI value of 76	Niyonzima et al. (25)	Tanzania	Mixed bred dairy cattle	Milk yield decline by 0.09 and 0.06 liters/day at THI value of 71–77	Ekine-Dzivenu et al. (74)	Kenya	Holstein, Jersey	Milk yield loss was estimated at 0.27, 0.18 and 0.35 kg/°C per day for the first, second and third lactation at THI value of 71	Mbuthia et al. (19)	South Africa and Namibia	Holstein	Milk yield decline at THI value above 72	Du Preez et al. (75)	Algeria	Holstein	Milk yield decline at −0.29 Kg/day per unit increase in THI value of 84	Ouarfli and Chehma (21)	Egypt	Crossbred Balady-Friesian	Higher RR and RT at THI values ranging from 79 to 90	Hady et al. (76)
Latin America	Brazil	Holstein, Purebred Gir	Milk yield decline at −0.094Kg/day per THI at THI value above 69 in 2015 and − 0.23 Kg/day per THI value above 66	Santana et al. (43), Santana et al. (77)			Milk yield decline at 0.5Kg/day per above 0.5 kg/ THI per day at THI value of 76	Santana et al. (78)			Estimated milk yield loss at THI = 74, was −0.106 kg/day per unit increase in THI	Negri et al. (79)		Crioulo Lageano, Curraleiro, Junqueira, Pantaneiro, Mocho Nacional, Indubrasil	Higher respiratory rates, sweating rates, and heart rates at THI Value from 70–90	Vieira et al. (80)	Mexico	Chinampo (*Bos taurus*), Holsteins and Jersey	Higher RT and RR at THI value of 72	Espinoza et al. (81)

## 4. Heat stress effects on milk and blood metabolites for dairy cattle breeds reared in tropical and sub-tropical countries

Heat stress induces modification of metabolites in the mammary glands of lactating dairy cattle, along with glycolysis, lactose, ketone, tricarboxylic acid cycle (TCA), amino acid, or nucleotide metabolism, which prevents the supply of substances for milk production in the mammary gland of lactating Holstein cows (82). Therefore, HS alters milk synthesis and composition by altering metabolisms of substances in the mammary gland tissues of lactating dairy cows.

Milk and blood metabolites are used to provide accurate information on the degree of HS in dairy cows as they change during HS (83). They also help in the genetic selection of genotypes and individual animals that are heat tolerant (82). These metabolites are identified in animal biofluids (e.g., serum, plasma, urine, and milk) through metabolomics approaches. A metabolomics approach is considered as potential method for extensive metabolite analysis of milk samples. Three analytical approaches are extensively used to study the milk metabolome: nuclear magnetic resonance spectroscopy (NMR), gas and liquid chromatography-mass spectrometry (GC–MS/LC–MS). Among these approaches, NMR can provide information on the direct association between metabolite richness and resonances.

LC–MS has greater sensitivity and reproducibility, together with a superior dynamic scale, becoming more worthy for the investigation and determination of non-volatile and greater macromolecular metabolites (84). Untargeted metabolomics applying LC–MS is a reliable method for the identification of a myriad of metabolite changes in a biological matrix, like blood, urine, cells, and tissues (85). On the other hand, proton nuclear magnetic resonance (NMR) combined with multivariate statistical analysis are very useful for metabolite profiling to investigate the metabolic variations in blood, milk and liver of heat stressed dairy cattle (86). Blood and milk metabolites (e.g., glucose, cholesterol, non-esterified fatty acids, blood urea nitrogen, creatinine, among others) which are identified through metabolomics are generally used to investigate the degree of HS in dairy cattle (51, 87). In the study conducted by Zheng et al. (54) at Heilongjiang province in China in Holstein dairy cows, seven blood metabolites including L-leucine, L-isoleucine, butanoic acid, and L-valine were significantly negatively associated with reactive oxygen species (ROS) while four milk metabolites, i.e., galactinol, undecane, cis-aconitate, ethanol were significantly positively correlated with ROS. The above metabolites are involved in the metabolic pathways associated with amino acid, carbohydrate, translation metabolism and metabolism of cofactors and vitamins. Both blood and milk metabolites were identified using GC–MS.

Using LC–MS and ^1^H-NMR, Tian et al. (88) in China, identified 53 potential diagnostic biomarkers for HS from milk samples of heat-stressed lactating Holstein dairy cattle. Included among them were lactate, pyruvate, citrate, creatine, proline, β-hydroxybutyrate, among others. The above metabolites are involved in various biochemical pathways including carbohydrate, amino acid, lipid and gut-microbiota-derived metabolism. Yue et al. (83) investigated the HS effects on milk and blood metabolites using proton nuclear magnetic resonance (^1^H-NMR) between HS groups and non-HS lactating Holstein dairy cattle in China and identified eight metabolites from milk samples and twelve metabolites from plasma samples which were significantly unrelated between the heat stressed and non-heat stressed-experimental groups. The identified metabolites include alanine, glycose, glutamate, urea, histidine, leucine, lipid, β-hydroxybutyrate, citrate, choline, formate, pyridoxamine, among others. The above metabolites are involved in different metabolic pathways including proteolysis, glycolysis, gluconeogenesis, lipolysis, galactose, purine and alanine metabolism, among others. Hu et al. (89) investigated the effects of HS on plasma metabolites between Inner-Mongolia Sanhe and Holstein cattle in China using ^1^H-NMR combined with multivariate statistical analyses. In their study, 26 potential diagnostic biomarkers for HS (valine, leucine, isoleucine, glutamate, glutamine, creatinine, among others) involved in amino acid, carbohydrate and lipid metabolic pathways were identified. Fan et al. (82) identified 33 potential metabolite biomarkers that can be used to identify the degree of HS in lactating Holstein dairy cattle in China. Among them glucose, lactate, β-hydroxybutyrate, creatinine, fumaric acid, and glycine were detected as indicative biomarkers for the degree of HS in lactating dairy cattle using LC–MS/MS. The afore-mentioned metabolites are involved in glycolysis, ketone, TCA, amino acid and nucleotide metabolic pathway. In this study, HS-persuaded changes in glycolysis-associated metabolites were detected for glucose, lactate, pyruvate, fructose 1,6-bisphosphate, among others (82).

In the subsequent study by Fan et al. (85), also conducted in China on Holstein dairy cattle, HS altered energy metabolism, including the decline in the concentrations of glucose, lactose, and galactose-1-phosphate, the increase in the concentrations of acetoacetate and β-hydroxybutyrate (ketone metabolites), and the alterations of TCA-associated metabolites (85). In their study, they identified 34 milk metabolites which are involved in amino acid, glycolysis, lactose, ketone, TCA, and nucleotide metabolic pathways (85). The alterations in nutrient splitting may impact the nutrient usage and the milk production in mammary gland. Findings from these studies indicate that identification of diagnostic biomarkers for HS can contribute to a better understanding of the biological mechanisms of responses to HS (89). This is very important for genomic selection of heat-adaptive dairy cattle and breeding programs aiming at improving heat tolerance in lactating dairy cows (82). Moreover, analysis of these metabolites can help to interpret the physiological mechanisms of HS-persuaded metabolic diseases and lactation (85).

On the other hand, HS alters blood parameters such as packed cell volume, white blood cells, red blood cells, hemoglobin, glucose, electrolyte concentrations, leukocytes, red cell distribution size, mean corpuscular volume, and platelets, among others (22, 72). For instance, neutrophilia happens in heat-stressed cattle owing to the collection of neutrophils from the bone marrow toward the portal blood (22). Red blood cell width (RDW) is not affected by elevation of THI and physiological parameters, implying that heat does not alter cell configuration and increased THI does not influence mean cellular hemoglobin concentration (MCHC) levels (22). Mean platelet volume (MPV) has been found to increase with THI and physiological traits (besides heart rate and panting score) (22). Kekana et al. (90) investigated the effects of HS on pregnant Jersey dairy cattle reared in Limpopo province of South Africa and found that at THI of 75–87, all lactating dairy cattle exhibited higher total protein, blood urea nitrogen and creatinine at 21^st^ and 75^th^ days of milk production. In the study by Jeelani et al. (72) conducted in the sub-tropical region of India on 33 crossbred dairy cattle of *Bos taurus* (Holstein Friesian) x *Bos indicus* (Hariana/Tharparkar), both red blood cells (RBC) and white blood cells (WBC) counts were significantly unrelated from those determined at THI of 72–74. Nevertheless, at THI of 76, the WBC counts were elevated while RBC counts reduced. The general trend showed that the rise in THI is correlated with elevation in WBC counts and decline in RBC counts. Gaafar et al. (52) investigated the effects of HS on blood parameters of Friesian suckling calves during winter and summer season in Egypt and found a significant effects of THI on different blood parameters. Generally, average trends of hemoglobin (HGB) concentration and count of white blood cells (WBC) and red blood cells (RBC) decreased significantly in hot season than in cool season as a result of HS.

Moreover, hematocrit percentage (HCT) and mean cellular volume (MCV) among other blood parameters increased significantly in hot season than in cool season as a result of HS (22). The decrease in WBC counts observed in the summer season might result from the body system’s response to stress stimuli after coming from the long winter season. In the study by Gao et al. (91) conducted on lactating Holstein cows in China, HS tended to decrease plasma glucose (8%) and non-esterified fatty acids (NEFA) (39.8%), and tended to increase blood urea nitrogen (BUN) (14.7%). Heat stressed dairy cattle showed reduced total plasma amino acids (17.1%), including some essential amino acids (Ile 42.5%, Lys 18.2%) and non-essential amino acids (Ser 10.7%, Arg 25.1%, Gly 20.8%, Cys 27%) (91). Rewe et al. (28) showed that under HS conditions, lactating dairy cattle exposed to increased temperature show elevated blood parameters including erythrocytes count, hemoglobin total concentration, and hematocrit, among others together with urinary pH and density, and the fecal dry matter content.

## 5. Genetic models and parameters for heat tolerance in dairy cattle breeds reared in tropical countries

The quantification of genetic effect relies on the estimates of additive genetic variance and genetic relationships between the traits in dairy cattle populations (38). Estimation of genetic parameters for heat tolerance are crucial for designing optimum breeding schemes and for predicting selection response. To characterize this relationship, the threshold of comfort and the production decline after a particular THI threshold have been used through different genetic models (92). Several studies have determined the genetic effect of HS on milk production traits and estimated the genetic components of HS in dairy cattle (47, 92–96). The statistical models used in the genetic evaluation studies applying test-day milk production records include (1) repeatability models which consider equal genetic relationship between every test-day milk records, (2) single trait and multiple traits models applying every test-day milk record as different parameters, and (3) random regression models (RRM) which treat a covariate function of repeated test-day milk records overtime (97). A more detailed description about these models was made in our previous review (45). The RRM have the potential to analyze every test-day milk records assuming that genetic and non-genetic variances change with days in milk (DIM), parity, lactation stage as do genetic and non-genetic relationships (97). The RRM also accommodate the additive genetic and permanent environmental variance within lactation (93).

Random regression models allow the estimation of genetic variance components and breeding values along the entire trajectory of a time-dependent such as days in milk (DIM) or environment-dependent descriptor like THI covariate (98). In RRM, every animal has two genetic effects, a steady effect that corresponds to animal performance in thermoneutral zone, and a HS effect corresponding to the rate of milk production decay under HS conditions (93). Modeling the effect of a breed as a function of time and environment allows the identification of GxE interactions via differences in genetic covariance components on various combinations of DIM over THI (46). On the other hand, optimization of genetic models that describe the genetic effects of HS on dairy cattle in tropical countries has been challenging because of a lack of proper animal recording. Traditionally, the broken line (BL) model has been used to describe the dairy cattle productive response to increased heat loads (47). This model assumes that production does not change within dairy cattle thermoneutral zone where no response to increasing temperature is observed and that after a particular breakpoint which indicates the beginning of HS, production declines linearly (47, 99). Since animal patterns of response to HS differ depending on climatic conditions, breed type (e.g., *Bos taurus* versus *Bos indicus*), production systems, lactation stage (lactating versus non-lactating), and physiological status of the animal, an alternative RRM fitting reaction norms and Legendre polynomial functions is considered rather than the BL model (45, 99).

Although interpreting RRM parameters biologically can be difficult, they are very useful for two reasons. First, they have higher flexibility to fit a regular pattern of decline and, second, they allow the application of eigen decomposition of the additive genetic covariance matrix to detect the selection criteria (92). Such criteria of selection are not associated between themselves and might support the genetic improvement of heat tolerance without having effect on degree of production (92). Moreover, in test day models used for selection on milk production traits, one of the eigen functions has been correlated with the persistence of lactations. This eigen function has been also recommended as a criterion for selection for this parameter to avoid the problems of antagonistic correlations between degree of production and other persistency measures (92). The above two approaches were named the splines (SP) model and the random regression reaction norm (RN) model based on Legendre polynomial (LP) functions, and have been applied to estimate the genetic parameters for heat tolerance in dairy cattle breeds reared in tropical countries. Negri et al. (79) applied RRM with LP functions of 3^rd^ order to milk production traits and included a model of THI in Brazil, and found a considerable genetic variance for heat tolerance. Table 4 summarizes information about studies that explored the genetic models and genetic parameters for heat tolerance in dairy cattle breeds reared in tropical countries.

**Table 4:** Genetic parameters for heat tolerance in dairy cattle breeds reared in tropical countries.

Region	Country	Dairy cattle breeds	Traits	Genetic model	Genetic parameters	Reference
Asia	Thailand	Holstein crossbreds	MY	Repeatability test-day model	Negative correlation for MY parameter between additive effects with and without HS across all three parities ranged between −0.21 and − 0.33. Negative correlation ranging from −0.45 to −0.56 was also detected between permanent environment effects.	Boonkum et al. (6)
			MY	ssGREML, repeatability model, RRM	Genetic correlation between MY and heat tolerance ranged from −0.06 to 0.13 for old data and 0.16 to 0.30 for recent data. Heritability (h^2^) estimates for MY ranged from 0.33 to 0.40 (recent data) and 0.19 to 0.21 for old data.	Sungkhapreecha et al. (5)
			MY, SCS, FPR	ssGBLUP, BLUP	Additive genetic effects observed were 0.094, 0.035, and 0.001, respectively for MY, FPR, and SCS using BLUP method. They also found 0.015,005, and 0.001 for MY, FPR, and SCS when used ssGBLUP. The h^2^ estimates ranged from 0.334, 0.049, and 0.060 for MY, SCS, and FPR.	Sungkhapreecha et al. (23)
Latin America	Brazil	Pure breed Gir	MY	RRM	A negative genetic correlation between general production level and specific ability to respond to HS was obtained (−0.23). A decline in additive genetic variance with rising THI values was observed for all DIM, especially between 40 to 200. A reduction in permanent environmental variance with increasing THI values was observed at any DIM. h^2^ estimates of up to 0.22 were obtained for lower THI values and DIM. h^2^ estimates of up to 0.29 were obtained for THI values <78 and DIM ˃ 290.	Santana et al. (43)
		Holstein	MY	RRM	Lower estimates of additive genetic variance observed for THI ≥ 70 and DIM 75–170. Lower estimates for permanent environment effects were obtained for greater values of THI and lower DIM. Higher h^2^ estimates ranging from 0.18 to 0.30 were obtained at THI ≤ 54 and DIM≤60. The genetic correlation between THI ≤66 and DIM 70–200 was 0.80.	Santana et al. (77)
		Holstein	MY, F%, P%, SCS	Two-trait RRM	The h^2^ estimates were greater for lower THI thresholds and longer DIM while h^2^ estimates for SCS increased with rising THI values in the 2^nd^ and 3^rd^ lactation. Genetic correlations between MY and THI values (THI of 47 and 80) were 0.90, 0.76; and 0.63 while genetic correlation between F%, P% and THI was 0.80.	Santana et al. (78)
		Guzerá	MY	RRM	At THI value from 72–90, h^2^ estimates for TDMY ranged from 0.16 to 0.35 throughout lactation and THI values.	Santana et al. (44)
		Holstein	MY	RRM fitting LP, SP, and Wilmink function	The h^2^ estimates for TDMY regressed with THI values ranged between 0.15 to 0.21 and 0.07 to 0.22 when using LP and SP functions, respectively.	Negri et al. (67)
			MY	RRM	Combining THI and DTV showed a rise in reliability of EBVs of sires with less than 30 daughters and a 27% increase for sires with 10 daughters.	Negri et al. (79)
			SFA, UFA, MUFA, PUFA, C16:0, C18:0, C18:1	Single-trait repeatability test-day models, RRM	Variations in h^2^ estimates between general and HS conditions increased from 0.03 to 0.06 for UFA parameters like unsaturated and monounsaturated. There was also a negative genetic correlation between general and HS additive genetic effects for saturated fatty acids ranging from −0.007 to −0.32.	Dauria et al. (4)
			MY, SCS, F%, P%, LP, CP, C16:0, C18:0, C18:1, SFA, UFA, MUFA, PUFA	Single trait animal model, RRM	The h^2^ estimate for milk production declined from 0.31 to 0.14, fat (0.28 to 0.16), protein (0.26 to 0.30), lactose (0.43 to 0.30), among other fatty acids profiles and continued to decline as THI increased from 51 to 78. Genetic correlation among breeding values at higher THI values was greater than 0.80 for all dairy cattle.	Carrara et al. (100)	Gir, Holstein, Gir x Holstein at F2	RT	GWAS	h^2^ and repeatability estimates for RT were 0.13 ± 0.08 and 0.29 ± 0.06, respectively.	Otto et al. (101)
Sub-Saharan Africa	Tanzania	Mixed breed dairy cattle	MY	FRM with RRM on a function of THI	Additive genetic variance for MY along THI spectrum ranged from 9.83 to 7.52. Covariance between the intercept and slope for additive genetic effect ranged from −0.24 to −2.10. There was also a negative genetic correlation (−0.06 to −0.09) between MY and THI values. h^2^ estimates for MY ranged from 0.38 to 0.50.	Ekine-Dzivenu et al. (13)

BLUP, best linear unbiased prediction; DIM, days in milk; DTV, diurnal temperature variation; EBVs, estimated breeding values; F%, fat percentage, P%, protein percentage; FRM, fixed regression models; FPR, fat to protein ratio; FP, percentage of fat; PP, protein percentage; LP, lactose percentage; CP, casein percentage; SFA, saturated fatty acids; UFA, unsaturated fatty acids; MUFA, monounsaturated fatty acids; PUFA, polyunsaturated fatty acids; C16:0, percentage of palmitic fatty acids; C18:0, percentage of stearic fatty acids; C18:1, percentage of oleic fatty acids; GWAS, genome-wide association studies; h^2^, heritability estimates; MY, milk yield; ssGBLUP, single-step genomic best linear unbiased prediction; ssGREML, single-step genomic restricted maximum likelihood; REML, restricted maximum likelihood; RRM, random regression models; LP, Legendre orthogonal polynomial functions; SP, Linear splines functions; RT, rectal temperature; SCS, somatic cell score; TDMY, test day milk yield; THI, temperature-humidity index.

Boonkum et al. (6) estimated the genetic effects of HS on milk yield traits using repeatability test models for Holstein crossbreds in Thailand. In this study, they observed a THI threshold ranging from 74 to 82. Sungkhapreecha et al. (5) used single-step genomic restricted maximum likelihood (ssGREML) and repeatability model and observed THI thresholds of 72–82. In a subsequent study, Sungkhapreecha et al. (23) used single-step genomic best linear unbiased prediction (ssGBLUP) and observed a THI threshold of 76. In this study, the genetic correlations between additive genetic effects were negative for all the traits using both methods. However, the positive additive genetic effects were observed for SCS for both methods.

In Brazil, Santana et al. (43) estimated the genetic parameters for milk yield traits using RRM and observed a HS threshold value of 69 with a MY loss estimated at −0.019 kg/year. In this study, it was observed that Gir dairy cattle population were becoming more susceptible to HS. They also found that smaller responses to selection are expected for combinations of THI and DIM values. In subsequent studies, Santana et al. (77) reported THI thresholds ranging from 54 to 79 and a genetic antagonism between milk production performance and HS resilience. This is an important aspect for tropical countries with a hot climate that aim to genetically select for higher milk production but also animals which are highly resistant to extreme temperature and humidity. Santana et al. (78) also used two-trait random regression animal models to estimate genetic parameters for MY, fat percentage (F%), protein percentage (P%), and SCS and observed a THI value of 76 as HS threshold. In this study, 53% of TD milk records were obtained at THI values above 66, and other 14% at THI values greater than 72. There was a decline in milk yield estimated at 0.5 kg/day per cow per THI. They also observed a reduction in MY, SCS, F % and P% at THI values ranging between 66 and 72. They also noted that response to selection can vary as a function of THI and DIM. In another study, Santana et al. (44) estimated the genetic parameters for MY records of Guzera cattle using RRM. In this study, they observed THI thresholds ranging from 72 to 90. Negri et al. (79) estimated the genetic components of MY for Brazilian Holstein cattle using RRM which fitted Legendre orthogonal polynomials, linear splines and Wilmink function. In this study, they obtained a THI threshold of 84 as HS threshold with an average milk yield/day of 23.95 ± 1.9 kg/per THI per cow for THI˃74. They observed that environmental variation increased as the additive genetic variation declined, this directly interfered the h^2^ estimates of MY trait.

In a subsequent study, Negri et al. (67) obtained a THI threshold of 74, associated with MY losses estimated at −0.106 kg/day/THI per cow. Diurnal temperature variation (DTV = 13) was associated with −0.45 kg/day of MY losses. They recommended that it is necessary to include the climatic variables in the genetic evaluation models for genetic selection of high performing sires as many sires could be penalized owing to their daughters being affected by HS. Dauria et al. (4) estimated the genetic parameters of milk fatty acids of Brazilian Holstein dairy cattle using single-trait repeatability test-day models and RRM. In this study, they observed a THI threshold of 68. Carrara et al. (100) estimated the genetic parameters for MY traits and fatty acids of Brazilian Holstein dairy cattle using single trait animal model and RRM. In this study, they obtained a THI threshold of 60. Also, they observed a small variation in the variance components over THI, indicating moderate to low HS for dairy cattle as a result of mitigation strategies using fans, sprinklers, shade trees, and other cooling devices applied at the farm. Otto et al. (101) estimated the genetic components of RT for Gir x Holstein crossbred dairy cattle using GWAS. In this study, they obtained a THI threshold of 97 (42°C and 60% RH). In Tanzania, Ekine-Dzivenu et al. (13) estimated the genetic components of MY for mixed breed dairy cattle using fixed regression model with RRM on a function of THI. In this study, they identified THI values ranging between 67–76. They found that genetic selection for higher milk production disregarding heat tolerance will lead to a deterioration of heat tolerance in this dairy cattle population. In conclusion, RRM fitting RN and LP functions are more suitable than other stated models during the genetic evaluation for milk production and composition traits over different THI thresholds in heat tolerance studies. The RN functions are more convenient to continuous environmental descriptors like THI as they help in the distinction allying individuals that are more or less affected by environmental variations (100). Additionally, through RN functions, it is possible to obtain two genetic components for each individual, i.e., general production capacity and specific capacity to respond to HS (77).

## 6. Genes responsible for heat tolerance in dairy cattle breeds reared in tropical countries

Understanding the mechanisms of climatic adaptation and heat tolerance of dairy cattle breeds reared in tropical countries is vital for the optimization of breeding schemes and conservation of genetic resources (102). Genetic variation in heat tolerance at physiological and cellular levels between *Bos indicus* and *Bos taurus* breeds reared in tropical countries has been estimated in various studies (103). Heat tolerance can be identified based on the expression or activation of particular biological biomarkers including heat shock transcription factor (HSF), heat shock proteins (HSP70, HSP90, and HSP27), and *Slick Hair* gene (36). HSF and HSPs known as molecular chaperons, are crucial genes associated with heat tolerance (37). They play an important cytoprotective role during cellular stress by avoiding the formation of non-functional proteins, maintaining protein folding, and preventing protein aggregation which result into protein homeostasis during cellular response to HS (37, 104). HSPs also activate the immune response mechanisms during HS (37). Heat stress causes misfolding of proteins at cellular level and a cell fails to perform DNA replication, transcription, polypeptide synthesis, and transport of materials (105). Given those HS effects, HSPs are activated and reduce accumulation of the denatured proteins in the cell and enhance its survivability and ability to overcome oxidative stress and thermal stress (105). Moreover, HSP70 act as molecular chaperons and play crucial roles in cellular thermotolerance, apoptosis, immune-modulation and heat tolerance (37, 105). HSP70 function as inducible chaperons and provide a second window of protection while stabilizing the native conformation of proteins and maintaining cell survivability during HS conditions (37, 106). An early induction of HSP70 is associated with the development of heat tolerance in dairy cattle (37). Moreover, HSP40 which harbors DNAJA1, DNAJB1, and DNAJA2 genes, regulates the ATPase activity of HSP70 through interaction with the J domain of the HSP70 proteins (104). Additionally, HSP40 and HSP90 function as co-chaperons of HSP70 and play important roles in the restoring of protein conformation after heat shock (106).

Studies have indicated that worldwide, there exists five continental groups of cattle, i.e., European taurine, Eurasian taurine, East Asian taurine, Chinese indicine and Indian indicine (107). Heat tolerance, ectoparasite resistance, low maintenance cost, and low susceptibility to the gastro-intestinal parasites are important physiological traits of Indicine over taurine (108, 109). The above physiological traits help *Bos indicus* breeds to grow under tropical and subtropical climates of Africa, South-east Asia, Brazil, Northern Australia, Southern China and parts of the USA (107, 108). The taurine breeds which have increased metabolic rate and feeds requirements are mainly reared in Europe, Northern America, and Australia (108).

To better understand the variation in HS response between Holstein-Friesian (taurine) and Sahiwal (indicine) in India, Kishore et al. (106) investigated the temporal profile of HSP40, HSP60, HSP70, and HSP90 candidate genes using qRT-PCR at 42°C. In their study, they observed that Holstein-Friesian was the most affected by the heat shock than Sahiwal, indicating that the latter is highly adaptable to HS conditions. In another study conducted in Brazil, Otto et al. (101) used GWAS through breed of origin alleles (BOA) to compare the HS response between Holstein and Gir dairy breeds. In their study, it was observed that the proportion of animals with HH and GG alleles were 2.9 and 49.9%, respectively for higher RT in Holstein and Gir, indicating that Holstein dairy cattle were more affected by HS than Gir animals (101).

In Nigeria, Morenikeji et al. (110) used RNA transcriptomic approach to compare HS response between indicine (white fulani and N’Dama) and taurine (Angus and Holstein) dairy cattle breeds and identified six genes (ASIP, MC1R, TYRP1, TYRP2, TYR, and ALOX12E) to be responsible for heat tolerance. These six genes were upregulated in indicine than in taurine breeds. In particular ALOX12E gene was found to be associated with water retention in indicine to avoid dehydration, an adaptive trait important for white fulani and N’Dama survivability in tropical climates (110). Furthermore, Onasanya et al. (111) indicated that HSP70 and HSP90 genes identified in West Africa zebu breeds are thermo-regulatory candidate genes which are less sensitive to thermal signals such as high temperatures. These heat shock protein genes are predominant in the west and east Africa zebu such as white Fulani, N’Dama, Sahiwal, Boran, and Ankole which constitute more than 90% of cattle population in Africa (111).

Selection signature analyses have the potential to identify specific genomic regions and candidate genes associated with adaptive or productive traits of different dairy cattle breeds reared in HS environments of tropical countries (40, 112, 113). Using selection signatures and copy number variations to differentiate *Bos taurus* and *Bos indicus* breeds, Jang et al. (114) identified 313 candidate genes under selection and adaptation. A higher copy number of HMGA2 gene was identified in *Bos indicus* than in *Bos taurus* breeds. They also identified DNAJC18 gene in indicine specific deletion than in taurine. The DNAJC18 candidate gene has been shown to play a significant role in tropical adaptation and heat tolerance of East African short horn zebu (114). At China-Myanmar border, Sun et al. (107) conducted a study on Lincang humped cattle and identified MED16, DNAJC8, HSPA4, FILIP1L, HELB, BCL2L1, and TPX2 as candidate genes responsible for heat tolerance and parasite resistance. In India, Saravanan et al. (36) studied the selection signatures between taurine breeds and indicine breeds and identified candidate genes responsible for immune response and heat tolerance. Among those genes identified include NCR3 (in Gir), ARID5A (in Ongole), HIST1H2BN (in Sahiwal), BEFB4 and DEFB7 (in Tharparkar) for immune response. Genes responsible for heat tolerance included HSPA1L and HSPA1B in Gir, DNAJB4 in Ongole and GRXCR1 in Tharparkar (36).

Genes such as OLA1, SP9, HSPA12A were associated with heat tolerance in all Indicine cattle breeds (Gir, Hariana, Kankrej, Ongole, Red Sindhi, Sahiwal, and Tharparkar) but none of these genes were identified in taurine breeds (36). The majority of candidate genes identified in taurine breeds were all related to milk production traits and included CACNA2D1, ADARB2, WDR37 and LARP4B (Ayshire), KCNIP4, SLIT2, ADAP2, and LTF (Brown Suiss), SYN3, TIMP3, RFK, ACACA, HNF1B, and SCAP (Guernsey), ADARB2, WDR37, LARP4B, ZMYND11, PRNP, and CDH4 in Holstein Friesian, and LPHN2, ADARB2, WDR37, and LARP4B in Jersey (36). The genes which were found to be associated with heat tolerance in Indicine cattle breeds can be used for molecular detection of dairy cattle breeds with potentialities for heat tolerance. Additionally, the candidate genes identified in various studies can serve as the basis to explore the use of indicine breeds in crossbreeding schemes in order to transfer their adaptation abilities and rusticity to taurine breeds (115). Table 5 provides examples of candidate genes responsible for heat tolerance that have been identified in different dairy cattle breeds reared in tropical countries.

**Table 5:** Candidate genes responsible for heat tolerance in dairy cattle breeds reared in tropical countries.

Country	Dairy cattle breeds	Identification method	Candidate genes	Functions	Reference
India	Holstein Friesian, Jersey	mRNA sequencing	HSF1, HSP70, HSP90, IL18, IFNγ, IFNβ, TNFα	Adaptation, heat tolerance, immune response	Velayudhan et al. (37)
	Sahiwal, Karan Fries, Ladakhi, Holstein Friesian, Jersey	qRT-qPCR	HIF-alpha, EPAS1, SP70, HSP27	Adaptation to HS conditions in cold and hot regions	Verma et al. (40)
	Sahiwal, Tharparkar, Gir, Ongole, Vechur, Kangayam, Hariana	Genome-wide scan for selection signatures	FAM19A2, RAB31P, BEST3, DGKA, AHCY, PIGU, PFKP	Immune response, metabolic pathway, glucose transportation and sugars, signaling pathways, cellular processes, cell division, glycolysis regulation and general adaptation to HS conditions	Dixit et al. (116)
	Hariana, Vrindavani	Genome wide scan	HSP90AA1, HSPB1, HSPD1, CALCOCO2, BAG2, HSP90AB1, HSPA4, MAPK3, ACOT7, HSPE1	Immune response, Heat tolerance	Sajjanar et al. (109)
China (Yunnan-Myanmar border)	Lincang	Whole genome sequencing	LIPH, IRAK3, GZMM, ELANE, MED16, DNAJC8, HSPA4 FILIP1L, HELB, BCL2L1, TPX2	Immune response, parasite resistance, HS resistance	Sun et al. (107)
Brazil	Girolando, Holstein, Gir x Holstein	Genome Wide Association Study (GWAS)	LIF, OSM, TXNRD2, DGCR8	Regulation of cell survival after apoptotic stimuli, protection of cells from oxidative stress and general adaptation to heat stress conditions	Otto et al. (101)
	Gir	Whole genome sequencing	AMH, KIRRELS, MAPK, CAMP, CATHL1, CATHL2, CATHL3	Regulation of cell proliferation, gene expression, cellular response to osmotic stress and heat shock, cell survival, apoptosis, tick resistance	Liao et al. (117)
	Caracu Caldeano, Crioulo Lageano, and Pantaneiro	Genome wide assessment of copy number variations	BOLA-DQB, BOLA-DQA5, CD1A, TRBV3–1, DEFB13, DEFB7, PRG3, ULBP21	Cattle adaptation to harsh climatic conditions, heat tolerance, immune response and ectoparasite resistance	Peripolli et al. (115)
	Criollo Limonero, Senepol, Carora, and Romosinuano	GWAS, Haplotype analysis, runs of homozygosity, and selection signatures	*SLICK hair*, PRLR, SKP2, SPEF2	Heat tolerance	Huson et al. (118)
Colombia	Caqueteño Creole	Selection signature	CMPK1, FOXD2, ULBP, KRTAP9-2	Response to stress, tick resistance, thermotolerance between zebu and taurine breeds	Toro-Ospina et al. (119)
Kenya	East Africa Short Horn Zebu	Signature of selection	DNAJC8, DNAJC18, HSPA9, HSPB9	Heat stress response	Bahbahani et al. (120)
Nigeria	White Fulani, Sokoto Gudali, Red Bororo, Ambala	qRT-PCR/HRMA	HSP70, HSP90	Adaptability to heat stress	Onasanya et al. (111)
Ethiopia	Afar, Arsi, Bagaria, Bale, Begait, Boran, Choke, Fogera, Goffa, Horro, Mursi, Ogaden, Semien, Sheko, Butana, Kenana, Ankole, Muturu, N’Dama, Gir, Angus, Holstein	Whole genome sequencing	CHADL, CTNNA, MZB1, GNAS, ENSBTAG00000017475, MED, THRA, NR1D1, IL18, RARA, KIT, PLA2G2A, ITPR2, IL18, TRAF3IP2, PRKCZ, TBX21, IL4, SOAT1STING1, DNAJC18, BAG6	Immune response, ion homeostasis, cell differentiation involved in immune response, coat color pigmentation and general adaptation to tropical environments	Terefe et al. (16)
Sudan	Kenana, Butana, Aryashai, Baggara, Gash and Fulani	Selection signatures	DNAJC18, HSPA9, LEMD3, MSRB3, TBC1D30, WIF1, MARCO, HOXC, ARHGAP26, IRAK3, NR3C1, FAAP20, MORN1, SK1, FAAP20, PEX10, PLCH2, PRKCZ, PLCH2, PRKCZ, RER1	Heat stress response, immune response, body size and conformation, glucose homeostasis, insulin signaling and fat metabolism all associated with heat tolerance in hot and arid environments	Tijjani et al. (121)
South Africa	Nguni, Angus, and Holstein	Genome-wide scan for selection signatures	KRT222, KRT24, KRT25, KRT26, KRT27, HSPB9, CYM, CDC6, CDK10	Immune response, adaptation to tropical environments	Makina et al. (122)

HSF1, Heat shock transcription factor-1; HSP70, Heat shock proteins-70; HSP90, Heat shock proteins-90; IL18, Interleukin-18; IFNγ, interferon gamma; IFNβ, interferon beta; TNFα, tumor necrosis factor alpha; HIF-alpha, Hypoxia-inducible factor 1-alpha; EPAS1, Endothelial PAS domain-containing protein 1; SP, specificity protein; FAM19A2, Family with sequence similarity 19; RAB31P, Ras-related protein Rab-31; BEST3, Bestrophin 3; DGKA, Diacylglycerol kinase alpha; AHCY, Adenosylhomocysteinase; PIGU, Phosphatidylinositol Glycan Anchor Biosynthesis Class U; PFKP, Phosphofructokinase; LIPH, Lipase member H-Homosapiens; IRAK3, Interleukin 1 Receptor Associated Kinase 3; GZMM, Granzyme M; ELANE, Elastase neutrophil expressed; MED16, Mediator complex subunit 16; DNAJC8, DnaJ heat shock protein family (Hsp40) member C8; FILIP1L, Filamin A interacting protein 1 like; HELB=DNA Helicase B; BCL2L1, BCL-2 protein family-like 1; TPX2, Targeting protein for Xklp2; LIF, Leukemia inhibitory factor; OSM, Oncostatin M; TXNRD2, Thioredoxin reductase 2; DGCR8, microprocessor complex subunit DGCR8; AMH, Anti-Mullerian Hormone; KIRRELS, Kirre Like Nephrin Family Adhesion Molecule 1; MAPK, mitogen-activated protein kinase 1; CAMP=Cathelicidin Antimicrobial Peptide; CATHL1, Cathelicidin-1; CATHL2, Cathelicidin-2; CATHL3, Cathelicidin-3; BOLA-DQB, bovine leukocyte antigen (*BoLA*)-DRB; CD1A, Cluster of Differentiation 1a; TRBV3–1, T Cell Receptor Beta Variable 3–*1*; DEFB13, Defensin Beta 113; PRG3, Proteoglycan 3; ULBP21= UL16 binding protein 21; SLICK hair, mutation in a prolactin receptor; PRLR, prolactin receptor; SKP2, S-Phase Kinase Associated Protein 2; SPEF2, Sperm Flagellar 2; CMPK1, cytidine monophosphate kinase 1; FOXD2, Forkhead Box D2; KRTAP9-2, Keratin Associated Protein 9–2; CHADL, Chondroadherin Like; CTNNA, Catenin Alpha 1; MZB1, Marginal Zone B And B1 Cell Specific Protein; GNAS, guanine nucleotide binding protein; ENSBTAG00000017475, ENSEMBL *gene* ID *ENSBTAG00000017475*; MED, mediator complex; THRA, Thyroid Hormone Receptor Alpha; NR1D1, nuclear receptor subfamily 1 group D member 1; RARA, Retinoic acid receptor alpha; KIT, receptor tyrosine kinase protein; PLA2G2A, Phospholipase A2 Group IIA; ITPR2, Inositol 1,4,5-trisphosphate receptor type 2; TRAF3IP2, TRAF3 interacting protein 2; PRKCZ, Protein Coding Protein Kinase C Zeta; TBX21, T-box transcription factor 21; SOAT1STING1, stimulation of interferon cGAMP interactor; BAG6, BCL2-associated athanogene cochaperone 6; LEMD3, LEM domain containing 3; MSRB3, methionine sulfoxide reductase B3; TBC1D30, TBC1 domain family member 30; WIF1, Wnt inhibitory factor 1; MARCO, Macrophage Receptor With Collagenous Structure; HOXC, homeotic; ARHGAP26, Rho GTPase activating protein 26; NR3C1, nuclear receptor subfamily 3 group c member 1; FAAP20, FA core complex associated protein 20; MORN1, MORN repeat containing 1; SK1, sphingosine kinase 1; PEX10, peroxisomal biogenesis factor 10; PLCH2, phospholipase Eta 2; PRKCZ, protein kinase c zeta; RER1, Retention In Endoplasmic Reticulum Sorting Receptor 1; KRT222, keratin 222; KRT24, keratin 24; KRT25, keratin 25; KRT26, keratin 26; KRT27, keratin 27; CYM, chymosin; CDC6, cell division cycle 6; and CDK10, cyclin dependent kinase 10.

## 7. Mitigation strategies for heat stress in dairy cattle in the tropics

Heat stress causes a substantial deleterious effect on dairy industry, including reducing growth, reproductive efficiency, and milk yield and quality. Thus, strategies to mitigate the effects of HS need to be developed. However, there is no single strategy that is used to mitigate the negative impacts of HS in dairy cattle farming. The following strategies can reduce the effects of HS in dairy cattle: (1) physical modification of the environment (shading, cooling), (2) genetic development of heat tolerant animals, and (3) improved feeding management strategies (65, 123–126). A combination of the above practices can enhance the production performances of lactating dairy cattle in hot and humid climates, especially in tropical and sub-tropical countries. It has been indicated that cooling technologies have a high positive impact on eliminating the adverse effects of high THI in dairy cows (1). However, those strategies do not completely alleviate the adverse effects of HS owing to animal-related factors such as genetics, hair coat and metabolic heat generated (127). As such, identification of dairy cattle resistant to high heat load and utilization of suitable mitigation strategies can lead to improvements in animal welfare and profitability (128).

### 7.1. Physical modification of environment

Common strategies for improving comfort of lactating dairy cattle and sustaining optimum milk yield during HS periods are mainly executed by physical modification of the production environment. Modification of the environment involve heat abatement technologies that alter the environmental conditions to prevent HS including misters, cooling pads and strategies that enhance heat exchange between lactating cattle and the environment like soakers (129). Management systems such as watering, shade, tunnels, sprinklers, and barns can enhance passive ventilation, improve body heat loss, decrease body temperature, and enhance dry matter intake (DMI) (90, 126, 128). Dry matter intake usually decreases during hot weather conditions, thus nutrient density of the diet have to be increased (126, 130, 131). Providing dairy cattle with shade when temperatures are above their thermal comfort level can potentially help producers to reduce water consumption by the cows. This is significant because the dairy industry has the second highest water footprint in animal production (132). The effectiveness of these technologies in cooling and altering thermal conditions in cows may vary (46). To mitigate the adverse effect of HS in smallholder dairy production systems, a combination of short term and long-term protective actions are necessary. In the short term, the aim should be to motivate smallholder farmers establish herd management and animal husbandry strategies that efficiently safeguard the animals from extreme heat. These heat mitigation strategies could incorporate use of tree shadings, hay or straw shades or roofing with ridged sheets decorated with deliberate coating on top to throw back sunlight and utilization of elevated ceilings in buildings to decrease temperature assimilated inside. Other measures include provision of sufficient feed and water consistently and improving the utilization of concentrate with minimum roughage within the limits of sustaining efficient rumen function during hot weather (74).

Evaporative cooling has been used successfully as a heat-abatement system for extended period of the year across the southwest of USA, but is less applied in the southeast, where there is low temperatures and high humidity (46). On the other hand, conductive cooling systems have the potential to preserve water, are better hygienically compared with evaporation-based cooling technologies, and provide successful HS relief compared to fan-based technologies (133).

Provision of shade to the animals exposed to the sun is a successful way to prevent immediate solar short wave radiation (134) and minimizes the problems of sunburn, particularly in animals with less pigmented skin (135). Lactating dairy cattle with access to shade have lower rectal temperatures because the reduced solar heat load enable them to better use sensible routes of heat loss (63). In addition, milk yield and composition improves when shade is provided (134). Anderson et al. (63) reported that dairy cattle under shade and cooled by fans and spray produced 2 kg/day more milk than the dairy cattle under shade alone. Provision of shade and sprinklers results in increased body weights, higher milk yields, milk fat, protein, lactose and lower rectal temperature and respiration rate (55, 132, 136, 137). Reis et al. (58) indicated that tree shade assisted to reduce HS by 7.3%. Wetting dairy cattle in rivers and ponds also is a successful way of dissipating thermal stress by evaporation and convection (135). The utilization of natural or artificial shade within the pasture production system saves livestock from extreme solar radiation and changes their radiation balance (58). Studies have shown that when a dairy cattle is provided with shade, it increases rumination time, increases milk production and quality, and decreases CBT compared with a dairy cattle in unshaded shelter (61). In Brazil, Reis et al. (58) evaluated the behavioral and physiological responses of Gyr and Girolando dairy cattle breeds reared in shaded and unshaded environments. In this study, at THI of 79 to 83, the rumination time of Girolando and Gyr dairy cattle was 1.7 times higher in dairy cattle reared under shade. Lying time was 23% longer in dairy cattle exposed to the sun. Girolando dairy cattle had 35% higher PS than Gyr dairy cattle regardless of the environment. The PS increased by 2.5 times during the afternoon compared with the morning. However, the effect of this high THI on Girolando and Gyr dairy cattle was reduced by shade from 2.5 to 5.2% (58).

### 7.2. Nutritional and feeding strategies for minimizing heat stress effects in animals

Sustaining sufficient feed intake is an important method to reduce milk production decline. Among the strategies to rise dietary nutrient intake is the provision of high-quality feed, feeding a lot of grain and utilization of supplemental fats (138). The provision of degradable starch such as cereal grains (e.g., wheat- grain and corn-grain) reduces the amount of heat produced during digestion (138). In Australia, Garner et al. (138) provided diets composed of wheat grain, corn grain, and alfa alfa hay to Holstein-Friesian dairy cattle exposed to a controlled climate chamber of 33°C and 50% relative humidity. In this study, dairy cattle offered corn grains had lower RR and greater feed intake. Decreasing the forage to concentrate ratio may also increase the ration that is absorbed to higher extent. However, feeding excessive concentrate may result in rumen disorders including acidosis and cows may cease feeding (130). Inclusion of sodium bicarbonate in the diet can assist the rumen to accommodate a greater amount of concentrate. Other feed additives that have been utilized under HS conditions to maintain rumen health from dietary changes are yeasts, which enhance fiber degradations, and fungal cultures and niacin, which enhances energy utilization (131). Providing yeast cultures such as *saccharomyces cerevisiae* and fibrolytic enzymes, e.g., cellulases and hemicellulases produced by fungi and bacteria stabilize rumen pH, improves fiber digestion and rise postrumen nutrient flow which lead to improved heat tolerance in dairy cows (130). Environmental and feeding modifications for increasing feed intake are specific to each production system and depend on their additional value for improving animal performance, fitness, and well-being compared with their costs (65). It is recommended to choose and feed fresh, palatable and high quality feed to the greatest extent possible and provide ingredients that have a high digestibility for livestock to reduce the heat increment through nutrient utilization into the animal (139).

### 7.3. Genetic selection for heat tolerant dairy cattle

The genetic development of a heat tolerant dairy cattle genotype, maintenance of higher milk production and reproduction performance in challenging environments has been a lifelong desire of researchers and cattle breeders (140, 141). Reduction of HS effects in dairy cattle breeds raised in low input production systems, especially in Sub-Saharan countries should rely on genetic selection of animals that are thermotolerant and have genetic ability to survive under stressful conditions. This strategy is more suitable and cost effective to improve dairy cattle production in tropical environments (4). Moreover, the gains made through genetic selection are cumulative and permanent (96). The following strategies are considered when genetically selecting for or breeding a thermotolerant dairy cattle breed: (1) choice of breed or cross for raising in the farm, as some genotypes are more heat tolerant than others, (2) introgression of genes that are associated with thermotolerance, and (3) using selection criteria connected with heat tolerance (99).

Identification of dairy cattle resistant to high heat load and their use in genetic improvement programs can lead to improvements in animal welfare and profitability of dairy enterprises (128). A heat tolerant animal is one that sustains thermal balance during HS conditions (125). In tropical countries, it is better to choose dairy cattle genotypes with *Bos indicus* genetic composition because of their relatively high adaptive capacities to HS. It is recommended that genetic selection for heat tolerance in tropical countries should rely not only on the capacity of dairy cattle to regulate body temperature but also on the sustainability of milk production traits during HS conditions (142). An alternative strategy for genetic improvement of heat tolerance with regard to milk production traits is to select for low milk production decline rather than for body temperature regulation during HS conditions (142).

Tropical and subtropical dairy cattle genotypes have a higher adaptive capacity to climatic stressors than exotic temperate breeds. Traits like heat tolerance, adaptability toward seasonal changes, lack of feed, tolerance on tick infestation and metabolic disorders, are worthwhile in tropical climates. The afore-mentioned traits are usually observed in tropical dairy cattle genotypes, and should be introduced in temperate breeds kept in the tropics which have higher production performances than the indigenous genotypes (143). Zebu cattle (*Bos indicus*), for example, possess heat tolerance genes and cattle deriving from zebu genotypes are highly efficient to control body temperature in response to HS in comparison with cattle from *Bos taurus* genotypes of European source (125). Zebu (*Bos indicus*) cattle are extensively acknowledged as adapted to tropical and subtropical climatic conditions that are prohibitive to *Bos taurus* cattle (144, 145).

Osei-Amponsah et al. (125) stated that modifications in herd genetics, and selecting genotypes with *Bos indicus* genetic composition may be suitable in developing thermotolerant dairy cattle breeds. For example, India, which is the world largest milk producer, has embarked on crossbreeding schemes to improve the milk yield from local genotypes and 90% of cattle population in the country are *Bos indicus*. Presently, the country has 22.1 million crossbred cattle population, composed of Holstein Friesian and Jersey crosses (144). Other studies have shown that the rectal temperature of a heat-stressed dairy cattle is heritable, and selection for this trait offers the possibility to improve thermotolerance of dairy cattle (15). The heritability estimates of rectal temperature in US Holsteins vary from 0.13 to 0.17. This level of heritability estimate implies that genetic selection for HS response is feasible (146).

An alternative genetic strategy to mitigate HS effects without crossbreeding is the genome editing technology. The polymorphisms associated with heat tolerance can be transferred from one genotype to another. The *SLICK* haplotype detected in heat tolerant genotypes reared in Central and South America lead to animals having short and fine hair and with a greater control of body temperature (147). The introgression of *SLICK* haplotype gene from cattle genotypes that are genetically highly resistant to HS can confer thermotolerance into temperate dairy genotypes that are adapted to cool climates (148). *SLICK* haplotype initially detected in Senepol cattle has now been introgressed into Holsteins to enhance their thermotolerance (149). *SLICK* haplotype Holstein cows are highly effective in controlling body temperature, and do not show noticeable decline in milk production during HS (125, 148).

Advances in the use of sequence data from phenomics, genomics, and transcriptomics technologies together with findings from gene expression studies have led to availability of accurate genomic breeding values that can be used in selection across breeds and generations. Data from gene expression or genome-wide association studies (GWAS) can be applied to further enhance the accuracy of selection. GWAS have frequently been applied to detect regions of the genome that have a potential impact on a trait of economic value in dairy production (125). Using GWAS through conditional analyses to precisely determine the association underlying heat tolerance and degree of milk production in Australian Holstein cattle, Cheruiyot et al. (11) identified potential candidate variants and genes associated with the neuronal system (ITPR1 and ITPR2) and neuroactive ligand-receptor interaction basis for heat tolerance (NPFFR2 and CALCR). This has given innovative discernment that can assist to develop breeding programs to mitigate HS. GWAS studies for rectal temperature during HS conditions in lactating Holstein cows has been undertaken to detect SNPs connected with thermotolerant genes and this could help genetic selection for adaptation to HS (125). Furthermore, using single-step genomic best linear unbiased prediction methodology to detect genomic regions and recognized candidate genes involved in milk production during thermoneutral and HS conditions, Sigdel et al. (15) identified candidate genes that elucidate the greatest additive genetic variances for milk production during HS conditions. In their study, one genomic site on BTA15 was found to be greatly correlated with the degree of milk production during HS for all the three parities. This site contains candidate genes including PEX16 and MAPK8IP1 that are involved in the cellular response to HS. The gene MAPK8IP1 is implicated in regulating cellular response to heat shock, which, sequentially enhances transcription activity of diverse HS responsive genes that regulate multiple functions, such as cell survival and cell proliferation.

Genomic selection (GS) has a comparative advantage as it allows young bulls (and heifers) to be selected based on their thermotolerance genomic estimated breeding values (GEBV) in addition to other parameters (150). Genomic selection applies genome-wide DNA markers to detect the effects of several mutations that cause alteration in a complex trait such as heat tolerance, and permits young bulls and heifers to be selected based on their GEBV, and thus, improving genetic gain (8). This strategy should be promoted in many tropical countries as it has shown success in developed countries. As dairy bulls are increasingly selected for breeding based on GEBV (for a range of traits) rather than progeny testing, establishing GEBV for heat tolerance is necessary if this trait is to be considered in selection decisions (150). Heat tolerance GEBV has been estimated with an accuracy ranging from 0.42 to 0.61 by applying high-density SNP markers (125). In 2017, for the first time, GEBV for heat tolerance were released in Australia (125). Genomic estimated breeding values for heat tolerance are reliable and using these values in selective breeding can improve genetic gain and increase profitability in the farm. Based on GEBV, it is possible to identify thermotolerant animals that are better suited to cope with the current and future climatic conditions (150).

Ravagnolo and Misztal (96) recommended genetic selection strategies to identify heat tolerant animals using the random regression models (RRM) that quantify the degree of HS based on climate information on the test day. This approach offers the possibility to identify the effects of genotype-by environment interactions. Selection signatures such as genes regulating anemia and feeding behavior in the trypanotolerant N’Dama cattle, coat color and horn development in Ankole cattle, and thermotolerance and tick resistance observed in African zebu cattle give alternative chances for genetic selection (125). Based on a question frequently posed whether it is better to select a dairy cattle to suit the system, or alter the system to suit a dairy cattle and as dairy farmers in tropical countries operate mainly using grazing systems, it is better to identify dairy cattle genotype that farmers believe will suit their production environment (99, 151, 152).

## 8. Conclusion

This review provides a comprehensive description of the genes and genetic parameters for heat tolerance in dairy cattle breeds reared in tropical countries. It highlights various indicators used to measure HS response in dairy cattle including environmental variables such as ambient temperature, relative humidity, solar radiation and wind speed. It also discussed decline in milk yield and alterations in milk composition traits such as fats, proteins, lactose, solids-not-fat percentages, and somatic cell score as indicators of HS. Additionally, physiological parameters such as core-body temperature, respiration rate, rectal temperature, panting score, drooling score, heart rate, and pulse rate as well as behavioral changes are potential indicators of HS in dairy cattle. This review describes various indicine and taurine dairy breeds reared in tropical countries and the candidate genes for heat tolerance that have been identified in tropical dairy cattle breeds. It discusses three approaches, namely gas and liquid-chromatography tandem mass spectrometry, GC-LC/LC–MS, and proton nuclear magnetic resonance (^1^H-NMR), used to identify milk and blood metabolites as vital biomarkers of HS in dairy cattle breeds, and the effects of HS on those metabolites.

The phenotypic response and genetic parameters for heat tolerance in dairy cattle breeds reared in Asia, Latin America, and sub-Saharan countries are also presented with various genetic models used for estimating genetic components for heat tolerance. The review emphasized the genetic antagonism between milk production traits and heat tolerance, indicating the need to consider the genetic sensitivity of potential dairy cattle breeds reared in tropical environments to avoid the adverse effects of HS. The review also highlighted the potential use of *Bos indicus* breeds, which harbor a large number of genomic regions and candidate genes responsible for climatic adaptation, for crossbreeding with *Bos taurus* breeds to improve milk production and genetic adaptation in tropical countries. Management practices for mitigating HS, such as environmental modifications and improved nutrition and feeding strategies, were discussed, with genetic selection for heat tolerance being recommended as a cost-effective approach.

In conclusion, the adoption of genetic selection for heat-tolerant animals, along with environmental modifications, improved nutrition, and crossbreeding of temperate dairy breeds with tropical breeds, is recommended for mitigating the impacts of HS in dairy cattle, particularly in tropical countries. Further research on candidate genes and genetic parameters associated with heat tolerance, as well as the development of appropriate genetic evaluation models, can contribute to the breeding of heat-tolerant dairy cattle breeds, ultimately improving their performance and resilience in the face of climate change. Crossbreeding of temperate breeds with local breeds that are resilient to HS is a quick way for improving thermotolerance of animals and thus, it is highly recommended in sub-Saharan Africa.

## Author contributions

VH conducted the research during his PhD studies and prepared the initial draft of the manuscript. AN, ZN, CE-D, GM, RM, and SC conceived the study idea, agreed on the content, and edited the manuscript. All authors contributed to the article and approved the submitted version.

## Funding

This study was funded by the Partnership for Skills in Applied Sciences, Engineering and Technology (PASET) through the Regional Scholarship and Innovation Fund (RSIF) awarded to VH to carry out doctoral studies at SACIDS Africa Centre of Excellence for Infectious Diseases, SACIDS Foundation for One Health, Sokoine University of Agriculture, Morogoro, Tanzania and International Livestock Research Institute (ILRI, Nairobi, Kenya). The funders had no role in study design, the decision to publish, or the preparation of the manuscript. The findings and conclusions of this study are those of the authors and do not necessarily represent the views of the funders.

## Conflict of interest

The authors declare that the research was conducted in the absence of any commercial or financial relationships that could be construed as a potential conflict of interest.

## Publisher’s note

All claims expressed in this article are solely those of the authors and do not necessarily represent those of their affiliated organizations, or those of the publisher, the editors and the reviewers. Any product that may be evaluated in this article, or claim that may be made by its manufacturer, is not guaranteed or endorsed by the publisher.

## Glossary

BL: Broken line model
BLUP: Best linear unbiased prediction
BOA: Breed of origin alleles
BUN: Blood urea nitrogen
C16:0: Percentage of palmitic fatty acids
C18:0: Percentage of stearic fatty acids
C18:1: Percentage of oleic fatty acids
CBT: Core-body temperature
CP: Casein percentage
DIM: Days in milk
DRC: Democratic Republic of Congo
DTV: Diurnal temperature variation
EBVs: Estimated breeding values
F%: Fat percentage
FPR: Fat to protein ratio
FP: Percentage of fat
FRM: Fixed regression models
GC–MS: Gas chromatography–mass spectrometry
GEBV: Genomic estimated breeding values
GxE: Genotype by environment interactions
GS: Genomic selection
GWAS: Genome-wide association studies
h^2^: Heritability estimates
^1^H-NMR: Proton nuclear magnetic resonance
HCT: Hematocrit
HGB: Hemoglobin
HR: Heat rate
HS: Heat stress
HSP: Heat shock proteins
LC–MS/MS: Liquid-chromatography tandem mass spectrometry
LP: Lactose percentage
LP: Legendre polynomial function
MCV: Mean cellular volume
MUFA: Monounsaturated fatty acids
MY: Milk yield
NEFA: Non-esterified fatty acids
P%: Protein percentage
PR: Pulse rate
PP: Protein percentage
PS: Panting score
PUFA: Polyunsaturated fatty acids
qRT-PCR: Quantitative real-time polymerase chain reaction
RBC: Red blood cells
REML: Restricted maximum likelihood
RH: Relative humidity
RRM: Random regression models
ROS: Reactive oxygen species
RR: Respiration rate
RNA: Ribonucleic acid
RN: Reaction norms
RT: Rectal temperature
SCS: Somatic cell score
SNPs: Single nucleotide polymorphism
SFA: Saturated fatty acids
SP: Splines model
SR: Sweating rates
ssGBLUP: Single-step genomic best linear unbiased prediction
ssGREML: Single-step genomic restricted maximum likelihood
Ta: Ambient temperature
TCA: Tricarboxylic acid cycle
TDMY: Test day milk yield
THI: Temperature-humidity index
UFA: Unsaturated fatty acids
UK: United Kingdom
USA: United States of America
WBC: White blood cells
